# Sexually dimorphic role for vasopressin in the development of social play

**DOI:** 10.3389/fnbeh.2014.00058

**Published:** 2014-02-28

**Authors:** Matthew J. Paul, Joseph I. Terranova, Clemens K. Probst, Elaine K. Murray, Nafissa I. Ismail, Geert J. de Vries

**Affiliations:** ^1^Neuroscience Institute, Georgia State UniversityAtlanta, GA, USA; ^2^Center for Neuroendocrine Studies, University of MassachusettsAmherst, MA, USA; ^3^Northern Ireland Centre for Stratified Medicine, University of UlsterUlster, UK; ^4^School of Psychology, University of OttawaOttawa, ON, Canada

**Keywords:** play fighting, social behavior, bed nucleus of the stria terminalis, paraventricular nucleus of the hypothalamus, juvenile, weanling, sex differences

## Abstract

Despite the well-established role of arginine vasopressin (AVP) in adult social behavior, its role in social development is relatively unexplored. In this paper, we focus on the most prominent social behavior of juvenile rats, social play. Previous pharmacological experiments in our laboratory suggested that AVP regulates play in a sex- and brain region-specific manner in juvenile rats. Here we investigate the role of specific AVP systems in the emergence of social play. We first characterize the development of play in male and female Wistar rats and then ask whether the development of AVP mRNA expression correlates with the emergence of play. Unexpectedly, play emerged more rapidly in weanling-aged females than in males, resulting in a sex difference opposite of that typically reported for older, juvenile rats. AVP mRNA and play were correlated in males only, with a negative correlation in the bed nucleus of the stria terminalis (BNST) and a positive correlation in the paraventricular nucleus of the hypothalamus (PVN). These findings support the hypothesis that AVP acts differentially on multiple systems in a sex-specific manner to regulate social play and suggest a role for PVN and BNST AVP systems in the development of play. Differential neuropeptide regulation of male and female social development may underlie well-documented sex differences in incidence, progression, and symptom severity of behavioral disorders during development.

## Introduction

Many neurodevelopmental disorders exhibit sex differences in incidence, progression, and symptom severity (Fombonne, 2009; Abel et al., 2010). This is particularly striking among disorders affecting social development such as autism spectrum disorders (ASD) and attention deficit hyperactivity disorder (ADHD), which are much more common in boys than in girls (reviewed in Rutter et al., 2003). Sex differences in neurobiological mechanisms that influence social development likely contribute to this differential vulnerability. Unfortunately, our understanding of these mechanisms is quite poor.

Social play contributes to the development of adult social and emotional skills (Pellegrini, 1988; Vanderschuren et al., 1997; Hol et al., 1999; Van den Berg et al., 1999) and is ideal for studying the neurobiology of social development. Notably, deficits in social play have been associated with ASD, ADHD, and schizophrenia (Alessandri, 1992; Jones et al., 1994; Jordan, 2003). The most common form of social play is play fighting, where participants engage in rough-and-tumble behaviors that resemble light-hearted wrestling (Bekoff and Byers, 1998). Play fighting in rats emerges around 18 days of age, peaks at 30–40 days of age, and declines thereafter (Panksepp, 1981). At its peak, males typically play more than females (Olioff and Stewart, 1978; Pellis et al., 1997), making play particularly valuable for studying sex differences in social development.

The neuropeptide arginine vasopressin (AVP) has been implicated in a number of adult social behaviors (reviewed in Caldwell et al., 2008). AVP may play an equally important role in social behavior during adolescence. Studies in our and other laboratories have demonstrated that AVP 1a receptor (V1aR) antagonists influence levels of play fighting in juvenile hamsters and rats (Cheng and Delville, 2009; Veenema et al., 2013), but the direction of these effects depends upon the sex of the subject and brain area injected. For example, intracerebroventricular injections of a V1aR antagonist decrease play fighting of male rats, but increase that of females, whereas septal injections cause the opposite effects (Veenema et al., 2013). It is not clear, however, which systems are responsible for AVP’s actions on social play.

AVP neurons in the bed nucleus of the stria terminalis (BNST) and paraventricular nucleus of the hypothalamus (PVN) are candidate sources for AVP’s actions on play. The lateral septum receives projections from the BNST (De Vries and Buijs, 1983), a primary node in the neural network thought to play a role in social behavior (Newman, 1999). Furthermore, AVP cells within this nucleus are sensitive to sex steroids and exhibit the most consistent sexual dimorphism found across vertebrates, with more cells in males than in females (De Vries and Panzica, 2006). In the PVN, AVP mRNA expression is altered by experimental manipulations that impact agonistic and affiliative interactions, including play (Veenema et al., 2006; Veenema and Neumann, 2009; Murakami et al., 2011; De Souza et al., 2013).

In the present study, we used a developmental paradigm to investigate a potential role for BNST and PVN AVP systems in the regulation of play. Hence, in a preliminary experiment, we characterized the development of play behavior in male and female Wistar rats. In a subsequent experiment, we tested whether the development of AVP mRNA expression in the BNST and PVN correlates with play’s emergence. Based on our previous psychopharmacological data, we predicted that the development of AVP expression in the BNST and PVN would correlate with the emergence of play behavior in sexually dimorphic and region-specific ways. As this was indeed the case, our data suggest distinct and sexually dimorphic roles for AVP cells in the BNST and PVN in play development. They also reveal a novel sex difference in the emergence of play.

## Materials and methods

### Animals and housing conditions

Wistar rats were purchased from Charles River Laboratories and housed in opaque plastic cages (48 × 27 × 20 cm) with Carefresh bedding and wood chips. Rats that arrived prior to weaning were housed with their mother and siblings. For all experiments, the day of birth was considered postnatal day 0. Room lights were set to a 12:12 light:dark cycle (lights off at 17:00 h ET), and ambient temperature was maintained at 23°C. Food and water were available *ad libitum*. All procedures were in accordance with the Guide for Care and Use of Laboratory Animals and were approved by the Animal Care and Use Committee at the University of Massachusetts, Amherst.

### Experimental procedures

#### Experiment 1a: emergence of play behavior

Pups arrived between 7–14 days of age with their mother and siblings (10 pups/litter consisting of 5 males and 5 females). Each pup was tested for play behavior once at 16, 19, 21, or 24 days of age. Testing each animal only one time ensured that effects seen in the current study were due to developmental changes rather than experience of repeated testing. Weanling-aged rats were removed from their litters and single-housed for 30–60 min before being paired with a similarly treated age-matched, same-sex rat in a clean cage for play testing. Two randomly selected animals in each litter (1 male and 1 female) were not tested. Sample sizes were *n* = 4 play pairs for all groups. One video of an 18-day-old female play pair was out of focus and could not be scored, reducing the sample size to *n* = 3 in this group.

#### Experiment 1b: play behavior of juveniles

To determine whether the sex differences in play emergence persist into the juvenile phase, a second set of rats treated identically to those in Experiment 1a were tested for play behavior at 35–36 days of age. Pups arrived between 7–14 days of age with their mother and siblings (10 pups/litter of 5 males and 5 females). Again, two randomly selected animals in each litter (1 male and 1 female) were not tested. Sample sizes were *n* = 4 per group.

#### Experiment 2: arginine vasopressin (AVP) expression during the emergence of play

Pups arrived between 7–14 days of age with their mother and siblings (10 pups/litter consisting of 5 males and 5 females). Each pup was tested for play behavior once at 18, 19, 20, or 21 days of age. To ensure that pups were paired with a motivated playmate and that their play behavior was not suppressed by an unwilling partner, each weanling-aged rat was tested with a juvenile (35 days of age) of the same-sex that had been single-housed for 24 h before testing; in rats, play peaks around 30–40 days of age, and 24 h of isolation further stimulates play (Panksepp, 1981). Pairing weanlings with a motivated juvenile stimulus rat also removed the need to isolate the experimental weanlings prior to the play test, a procedure that would confound measures of AVP expression. Weanling-aged rats were taken directly from their litters and placed in the juvenile’s home cage. Sample sizes were *n* = 8 for male and female 18-day-old rats, *n* = 7 for male and female 19-day-old rats, *n* = 4 for male and female 20-day-old rats, *n* = 5 for male 21-day-old rats, and *n* = 4 for female 21-day-old rats. At the end of the 30-min play test, a subset of weanling-aged rats was sacrificed under red light by CO_2_ inhalation (*n* = 5 for 18-day-old females, 18-day-old males, and 21-day-old males; *n* = 4 for 21-day-old females). Brains were removed, immediately frozen in 2-methylbutane that was kept on dry ice, and stored at −80°C until processed by *in situ* hybridization for AVP mRNA expression.

### Play behavior tests

All play tests were conducted within the first 2.5 h of lights off under red light. Animals were paired with a same-sex playmate in similar cages as used for housing. Each play session lasted for 30 min, during which behaviors were recorded using the nightshot setting on a Sony Handycam video camera (DCR-SR85). The number of pounces (lunges toward the nape of the playmate’s neck), pins (animal on top, holding playmate in a supine position), and boxing events (both animals standing on their hind paws and pushing each other with their forepaws), as described in Meaney and Stewart (1981b) and Vanderschuren et al. (1997), were scored by a researcher blind to the treatment conditions using JWatcher software.^1^

### Arginine vasopressin (AVP) *in situ* hybridization and analysis

Brains collected from weanling-aged rats were cut on a cryostat into 20 µm thick coronal sections, thaw-mounted onto Superfrost/plus slides (Thermo Fisher Scientific, Pittsburgh, PA), and stored at −80°C. Every third section was processed for AVP *in situ* hybridization. These sections were postfixed in 4% paraformaldehyde at 4°C for 15 min and then rinsed in the following buffers: (1) 0.1 M phosphate-buffered saline (pH 7.4) for 2 min at 4°C; (2) 0.1 M triethanolamine buffer (pH 8.0) for 1 min at room temperature (remainder of rinses were done at room temperature); (3) 0.1 M triethanolamine buffer (pH 8.0) with 2.5 µl/ml acetic anhydride for 10 min; (4) 2X standard sodium citrate (SSC) buffer (0.3 M sodium chloride and 0.03 M sodium citrate in distilled water, pH 7.0) for 1 min; (5) 70% ethanol for 2 min; (6) 100% ethanol for 2 min; (7) chloroform for 5 min; and (8) 100% ethanol for 2 min. *In situ* hybridization was carried out using a mixture of two oligodeoxyribose antisense probes. Probe 1: 5′-CAGCTGGCTG GGACACAAGAGTCCGTGGATTCTGCCAAGCCCCGGGTC-3′ Probe 2: 5′-CCGCGCTCGGGAGCAGAGCAACGCCACGCAGC TGGACGG-3′. Probes were labeled at the 3′ end with ^35^S-dATP (PerkinElmer, Waltham, MA) using terminal deoxynucleotidyl transferase (T4427, Sigma, Saint Louis, MI) and terminal transferase buffer (B0315S, New England BioLabs, Ipswich, MA). For hybridization, 100 µl of ^35^S-labeled probe in hybridization buffer (50% formamide, 10% dextran sulfate, 0.3 M NaCl, 10 mM Tris, 1 mM EDTA, 1X Denhardt’s solution, 10 mM dithiothreitol) was added to each slide. Slides were then coverslipped and incubated at 37°C overnight. All solutions for the pre-hybridization steps were made with Diethylpyrocarbonate (DEPC)-treated water. Following hybridization, coverslips and hybridization buffer were removed by dipping in 1X SSC at room temperature. Slides were rinsed in 1X SSC for 4 × 15 min at 55°C. Subsequent rinses were done at room temperature in the following order: (1) 1X SSC for 15 min; (2) 1X SSC for 2 × 45 min; (3) 70% ethanol for 2 min; (4) 95% ethanol for 2 min; and (5) 100% ethanol for 2 min.

Hybridization signal was visualized using two methods. First, slides were exposed to film. Second, slides were dipped in emulsion (NTB2, Kodak, Rochester, NY) at 42°C under safelight and stored at 4°C in light tight boxes containing desiccant capsules. Slides were developed under safelight in the following solutions: (1) Dektol-19 developer (1:1 with purified water) for 2 min at 15°C; (2) distilled water for 30 s; (3) fixative (Kodak) for 5 min; and (4) distilled water for 5 min. Slides were then rinsed in running water for 3 min, lightly counterstained with 2% methyl green for 30 s, rinsed with running water until clear, dehydrated in 50% ethanol, and coverslipped with Cytoseal 60 (Richard-Allen Scientific, Kalamazoo, MI).

AVP mRNA expression in the BNST and PVN was analyzed using the exposed film. The film was placed on a lightbox and the full rostro-caudal extent of each nucleus was photographed. Radioactive label, indicating the presence of AVP mRNA, was quantified as the number of pixels above background using the threshold function of ImageJ software (NIH, Bethesda, MD) and summed for all sections containing the BNST or PVN. The radioactive signal from AVP expression in the PVN was much greater than that in the BNST, and appeared overexposed in the majority of cases. Therefore, AVP expression in the PVN was also quantified from the emulsion-dipped slides; BNST AVP expression was not quantified using this method because the clusters of silver grains in this nucleus were difficult to detect above background in the weanling-aged rats. The full rostro-caudal extent of the PVN on the right side of the brain was photographed using a SPOT camera and software on a Nikon Eclipse E6200 microscope. Several pictures were often needed to capture the entire PVN at this magnification. These pictures were assembled into a single TIFF file using Adobe Photoshop. Radioactive label was quantified as the number of pixels above background using NIH ImageJ and summed for all sections containing the PVN.

### Data analysis

Play behavior and AVP expression were analyzed using a two-way ANOVA or Student’s *t*-test. *Post-hoc* comparisons were conducted using Fisher’s PLSD only when the overall ANOVA was significant. Correlations between play behavior and AVP expression were assessed by simple regression analysis. For play behavior, the number of pounces, pins, and boxing events were analyzed separately and combined as the sum total of play behaviors that occurred during the play session (Total Play Score). The unit of analysis was the pair. Therefore, a single score was provided for each age-matched pair in Experiment 1a and b, and only the behavior of the weanling-aged rat was considered in Experiment 2 (behavior of juvenile stimulus animal was not included in the analyses). This yielded play scores derived from differing numbers of individuals: two rats per play pair in Experiment 1a and b, but only one rat in Experiment 2—because behavior of juvenile rats was not included in analyses. Therefore, scores from Experiment 1a and b were converted to the mean number of behaviors per playmate to normalize play scores across experiments. All statistical analyses were conducted using Statview 5.0.1 (SAS Institute, Cary, NC). Significance was assumed if *P* < 0.05.

## Results

### Experiment 1a: emergence of play behavior

**Figure 1:**
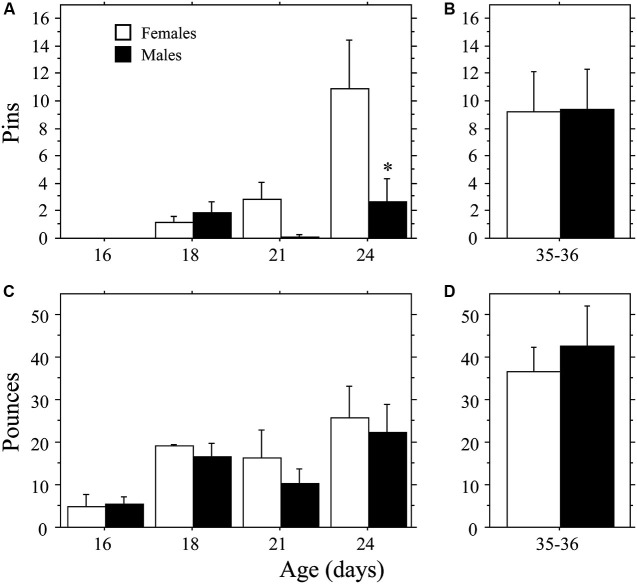
**Development of play behavior in female and male age-matched rat pairs**. Mean number (+s.e.m.) of pins **(A** and **B)** and pounces **(C** and **D)** per playmate in weanling (**A** and **C**; Experiment 1a) and juvenile (**B** and **D**; Experiment 1b) play pairs; * indicates significant sex difference within age group.

Female weanling-aged rats exhibited a more rapid emergence of pinning behavior than males (Figure 1A) resulting in a sex difference opposite that typically reported for juveniles (e.g., Krebs-Kraft et al., 2010). Overall, females displayed more Pins than did males (main effect of sex, *P* < 0.03, two-way ANOVA). The number of Pins increased across age (main effect of age, *P* < 0.002, two-way ANOVA), but only in females (interaction, *P* < 0.04, two-way ANOVA). At 24 days of age, females displayed more Pins than did males (*P* < 0.0009, Fisher’s PLSD). Total Play (data not shown) and Pounces (Figure 1C) increased across age (main effect of age, *P* < 0.005 for both measures, two-way ANOVA). However, there was no effect of sex and the interaction was not significant (*P* > 0.05 for both measures, two-way ANOVA). Boxing was low and did not differ across age or between sex (age, sex, interaction, *P* > 0.05; two-way ANOVA; data not shown).

### Experiment 1b: play behavior of juveniles

The higher levels of pinning seen in female weanlings compared to male weanlings did not persist into the juvenile stage of development. There were no differences in Total Play, Pins, Pounces, or Boxing between male and female juvenile rats (*P* > 0.32 for all measures, Student’s *t*-test; Figures 1B, D; Total Play and Boxing data not shown).

### Experiment 2: arginine vasopressin (AVP) expression during the emergence of play

#### Play behavior

**Figure 2:**
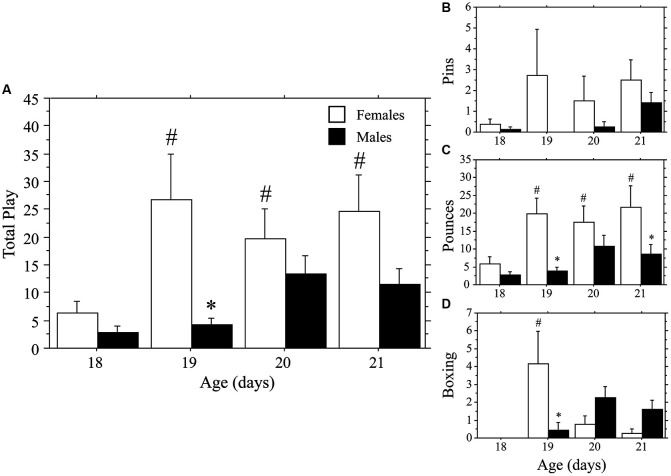
**Play emerges more rapidly in female than in male rats**. Mean number (+s.e.m.) of all play behaviors **(A)**, pins **(B)**, pounces **(C)**, and boxing events **(D)** in weanling-aged females (open bars) and males (closed bars) paired with 35-day-old juvenile play partners; * indicates significant sex difference within age group; # indicates significant increase above day 18 baseline measure for females.

Both weanlings and juveniles exhibited the characteristic play behaviors in their natural forms. Only levels of Pins were affected by the differing ages of the playmates, which were variable and low in number in the weanlings. Similar to that seen in Experiment 1a, play emerged more rapidly in females than in males. This time, however, the effect was stronger for Total Play and Pounces than it was for Pins, which appeared to be hindered by the large body size of the juvenile stimulus playmate. No main effects or interaction were seen for Pins (main effect of age, *P* > 0.4; main effect of sex, *P* > 0.09; interaction, *P* > 0.60, two-way ANOVA; Figure 2). Female weanlings exhibited higher levels of Total Play and Pounces than did males (main effect of sex, *P* < 0.002 for both measures, two-way ANOVA; Figure 2). There was an overall increase in Total Play, Pounces, and Boxing events across age (main effect of age, *P* < 0.008 for all 3 measures, two-way ANOVA). Only females exhibited striking increases in Total Play, Pounces, and Boxing events between 18 and 19 days of age (*P* < 0.001 for all 3 measures, Fisher’s PLSD), which led to significantly higher levels in females compared to males at this age (*P* < 0.002 for all 3 measures, Fisher’s PLSD). While Pins displayed the same pattern, the overall ANOVA was not significant, prohibiting *post-hoc* comparisons. For males, play behaviors failed to significantly rise above day 18 values throughout the test period (day 18–21; *P* > 0.05 for Total Play, Pounces, and Boxing, Fisher’s PLSD).

#### Arginine vasopressin (AVP) expression at 18 and 21 days of age

**Figure 3:**
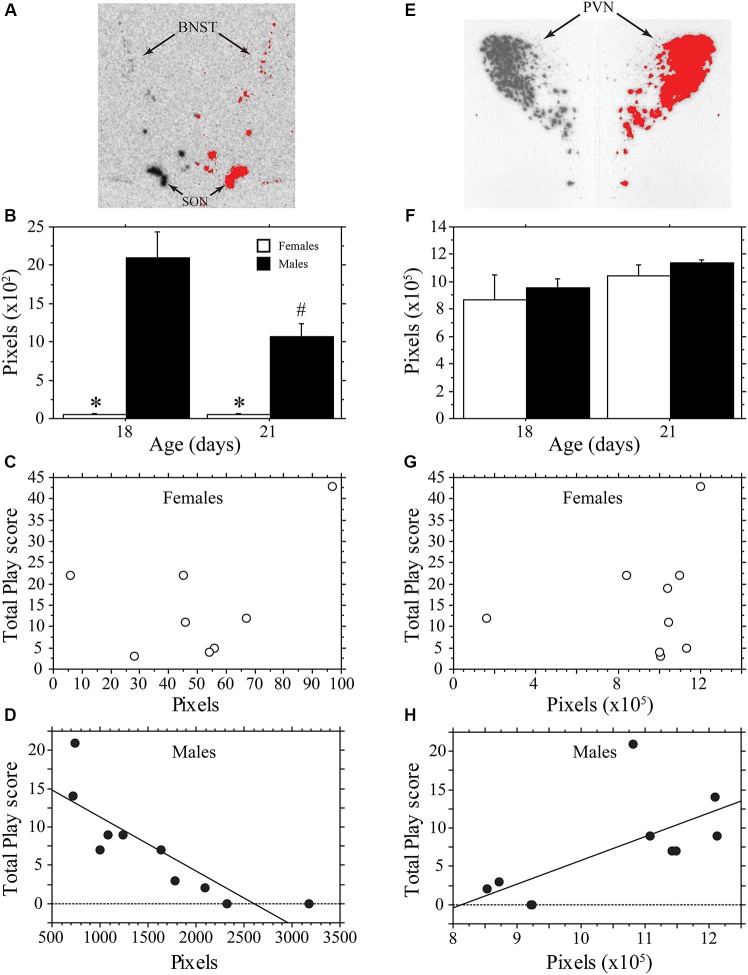
**AVP mRNA is correlated with play behavior of male, but not female, rats across development**. Representative *in situ* hybridization for AVP mRNA in the bed nucleus of the stria terminalis (BNST; **A**) and paraventricular nucleus of the hypothalamus (PVN; **E**); AVP mRNA expression on the left side of the picture is mirrored on the right with thresholding added in red. Mean number of pixels (+s.e.m.) of AVP mRNA in the BNST **(B)** and PVN **(F)** in male and female 18- and 21-day-old rats. Panels **C, D, G, and H** illustrate the correlational analysis of Total Play behavior and AVP mRNA in the BNST **(C, D)** and PVN **(E, H)**. Note the significant negative correlation with the BNST of males **(D)**, the significant positive correlation with the PVN of males **(H)**, and the absence of a significant correlations in females **(C, G)**. * indicates significant sex difference within age group; # indicates significant decrease in AVP mRNA of 21-day-old males from 18-day-old values.

Figure 3 contains representative pictures of the AVP in situ hybridization in the BNST **(panel A)** and PVN **(panel E)**. As expected, AVP mRNA expression in the BNST of weanling aged-rats was much higher in males than in females (main effect of sex, *P* < 0.0001, two-way ANOVA; Figure 3B). AVP mRNA varied across age (main effect of age, *P* < 0.04, two-way ANOVA) in a sex-specific manner (interaction, *P* < 0.04, two-way ANOVA). Specifically, expression declined in males between 18 and 21 days of age (*P* < 0.004, Fisher’s PLSD), but did not change in females (*P* > 0.99, Fisher’s PLSD). Thus, as play increased across development, AVP expression in the BNST of males decreased. To determine whether AVP mRNA levels were related to individual variations in play, we determined whether there was a correlation between the AVP expression of individual animals and their Total Play scores. We first examined both ages combined. Not surprisingly, the consistently low levels of BNST AVP expression in females was not correlated with Total Play (*P* > 0.28, Regression; Figure 3C). In males, however, there was a significant negative correlation between these variables that accounted for 71% of the variance (*P* < 0.003, *R*^2^ = 0.71, Regression; Figure 3D). Notably, this negative correlation remained significant when the analysis was restricted to 18-day-old male rats even though the sample size was small (*n* = 5; *P* < 0.05, *R*^2^ = 0.78, Regression); for 21-day-old males, the correlation fell short of significance (*P* = 0.16, Regression).

In contrast to the BNST, the PVN showed similar AVP expression between males and females, which did not vary significantly with age (main effect of sex, *P* > 0.4; main effect of age, *P* > 0.11; interaction, *P* > 0.98, two-way ANOVA; Figure 3F). AVP expression in the PVN of females was not significantly correlated with Total Play scores (*P* > 0.58, Regression; Figure 3G). In males, however, AVP expression in the PVN was positively correlated with Total Play (*P* < 0.04, *R*^2^ = 0.43, Regression; Figure 3H). The correlation was not significant when restricted to only 18- or 21-day-old males (Day 18, *P* = 0.07; Day 21, *P* > 0.62, Regression).

### Discussions

While AVP has been implicated in juvenile social play (Cheng and Delville, 2009; Veenema et al., 2013), the circuits through which AVP influences play are not known. Here we assessed AVP expression in the PVN and BNST during the emergence of social play in rats and found sex- and region-specific correlations between AVP mRNA expression and play behavior. These data are consistent with opposing roles for AVP neurons in the PVN and BNST in the regulation of play in males and suggest how AVP systems might interact to regulate the development of play in a sexually dimorphic manner.

As play behavior develops, AVP mRNA expression in the BNST of males declined, suggesting an inhibitory role for AVP in this nucleus. Indeed, we found a striking negative correlation between BNST AVP mRNA and play behavior in males. These data are consistent with our earlier observation that play behavior is increased in juvenile male rats infused with a V1aR antagonist into the septum (Veenema et al., 2013) and identify the BNST as the likely source of AVP’s inhibitory actions in the septum of males. In that same study, intracerebroventricular infusions of this V1aR antagonist, which reach a much wider range of potential neural AVP targets, reduced play in males. This suggests that AVP acts on multiple systems, with both stimulatory and inhibitory actions on play. Our data point to the PVN as a potential source of the stimulatory effect, as AVP mRNA in the PVN correlated positively with Total Play in males. This finding is also consistent with increased PVN AVP mRNA expression that accompanies enhanced offensive play behaviors in juvenile male rats after early postnatal maternal separation (Veenema and Neumann, 2009). Our findings could reflect regulation of AVP expression by development, the environment, or both. Weanlings were sacrificed 30 min after introduction to an unfamiliar 35-day-old juvenile, which might be sufficient time for environmental factors to alter AVP mRNA—restraint stress increases AVP mRNA in the PVN within 15 min (Dent et al., 2000). Nonetheless, our findings when considered with those discussed above suggest that different AVP systems have distinct influences on male play early in development, perhaps with BNST and PVN AVP systems opposing each other.

We did not detect a correlation between play and AVP mRNA in either the BNST or PVN of female rats. Nonetheless, AVP does influence play in this sex, at least during the juvenile phase, as infusions of a V1aR antagonist alter levels of play in females, again in a region-specific manner (Veenema et al., 2013). Effects of these infusions, however, are opposite to those seen in males. Hence, AVP influences play in males and females through distinct mechanisms, which might explain why we did not find significant correlations in females. AVP may act via other nuclei not considered in the present study that likely contribute to AVP’s actions on play, e.g., nucleus circularis, supraoptic nucleus, medial amygdaloid nucleus (Cheng et al., 2008; Kurian et al., 2008; Forbes-Lorman et al., 2012). Alternatively, AVP’s actions in females may be regulated at a different stage (e.g., peptide production or secretion). Given the high level of AVP expression in the PVN, it remains possible that AVP’s actions on play behavior in females are mediated through a subset of PVN cells (e.g., centrally projecting parvocellular cells; Sawchenko and Swanson, 1982) in which mRNA differences might be obscured when the PVN is analyzed as a whole.

As in previous studies, play first emerged around 18–19 days of age (Bolles and Woods, 1964; Panksepp, 1981; Thiels et al., 1990). Unexpectedly, play emerged more rapidly in females than in males, resulting in a sex difference (females > males) counter to that traditionally reported in juvenile rats (e.g., Meaney and Stewart, 1981b). This “reversed” sex difference did not persist beyond the weanling period, as sex differences were not detected in the 35-day-old juvenile rats tested under the same conditions (Figure 1). These data suggest that sex differences in play depend on the developmental stage at which animals are tested. Environmental factors are also likely to affect sex differences in play emergence. Detection of sex differences in play is highly sensitive to environmental conditions (Thor and Holloway, 1984). While males are consistently found to play more than females when tested in mix-sex groups (Meaney and Stewart, 1981b; Olesen et al., 2005; Parent and Meaney, 2008), this difference is often not detected in experiments that test animals in same-sex pairs (e.g., Panksepp and Beatty, 1980; Panksepp, 1981; Thor and Holloway, 1984; Veenema et al., 2013; present results). Thus, as with the traditional sex difference (males > females), the more rapid emergence of play in females may not manifest under all testing conditions. For example, using the mixed-sex testing paradigm, Meaney and Stewart (1981a) found higher levels of play in males compared to females when data were collapsed across 21–25 days of age (onset for each sex, however, was not reported). Nonetheless, we found the “reversed” sex difference in two experiments, even though they were conducted with different experimental protocols (e.g., duration of separation prior to testing, age of playmate, and neutral-testing-cage vs. resident-intruder paradigm). Future studies are needed to determine whether the more rapid development of play in females is restricted to play behavior, or whether it reflects a more general sex difference in overall development.

Early-life experience can also impact later levels of juvenile play in a sex-dependent manner (Parent and Meaney, 2008). Hence, it is possible that the stress of shipping in the second week of life contributed to the earlier emergence of female play, perhaps by selectively decreasing play in males. However, various early-life manipulations can increase or decrease play of juvenile males (Veenema and Neumann, 2009; Edelmann et al., 2013), or affect both sexes equivalently (Karkow and Lucion, 2013). No studies have directly tested shipping effects on play’s emergence.

It is tempting to speculate that inhibitory actions of AVP from the BNST are responsible for slower play development in males. At the end of the third week of life, BNST AVP mRNA is high in males, but virtually undetectable in females (present results; Szot and Dorsa, 1993). Likewise sex differences in AVP immunostaining in the lateral septum are similarly extreme at this age (De Vries et al., 1981). Therefore only males would be subject to this inhibition, and AVP’s decline around weaning (present results; Szot and Dorsa, 1993) may facilitate play development in this sex. Szot and Dorsa (1993) reported a similar decline in BNST AVP mRNA at 21 days of age from peak levels occurring around 7–14 days of age. Given that AVP mRNA is tightly regulated by testosterone (Miller et al., 1989, 1992), this transient peak in BNST AVP mRNA is likely the result of activation from the neonatal surge in testosterone that occurs around birth (Szot and Dorsa, 1993 and references therein). Further studies are needed to test whether this sexually dimorphic developmental regulation of BNST AVP has consequences for sex differences in social development, including play’s emergence.

While AVP mRNA expression in the BNST correlates negatively with the emergence of play in individual weanling-aged males (present results), prenatal exposure to an immune challenge, lipopolysaccharide (LPS), decreases both play and BNST AVP mRNA of juveniles male rats (Taylor et al., 2012). There are several potential explanations for these seemingly contradictory findings. First, the role of BNST AVP in play behavior may change across age, similar to that found for septal AVP in social recognition in juveniles compared to adults (Veenema et al., 2012). Taylor et al. tested juveniles, whereas we tested weanlings. Perhaps AVP’s role differs in the onset vs. maintenance of play. Second, LPS injections might have reduced a stimulatory drive to play, which could have been accompanied by a compensatory reduction in inhibitory mechanisms in the BNST. Collectively, these studies indicate a complex role for AVP in social development that differs by age and sex.

Neurodevelopmental disorders that impact social behavior often show striking sex differences in incidence, progression, and symptom severity (Rutter et al., 2003; Fombonne, 2009; Abel et al., 2010). Understanding the underlying mechanisms that direct social development, particularly those mechanisms that differ between males and females, may explain sex differences in vulnerability to neurodevelopmental disorders (De Vries, 2004). Evidence is now mounting that social and affective disorders involve perturbations in AVP and other neuropeptide systems (e.g., Heinrichs et al., 2009; Harony and Wagner, 2010). The present investigation implicates the PVN and BNST AVP systems in AVP’s sexually dimorphic actions on play, hints at a previously unknown sex difference in social development, and suggests that AVP is a regulating factor in the emergence of play. Findings from multiple studies, including our own, demonstrate that AVP acts in a sex-specific manner during development, as it does in adulthood.

### Author contributions

Matthew J. Paul and Geert J. de Vries designed the study, participated in the behavioral and neuroanatomical analysis, and wrote the first and subsequent drafts of the paper. Matthew J. Paul, Joseph I. Terranova, Clemens K. Probst, Elaine K. Murray, and Nafissa I. Ismail assisted in developing the protocol for, and execution of, behavioral testing and *in situ* hybridization. All authors discussed the results, contributed to the writing of the paper, and approved its final version.

## Conflict of interest statement

The authors declare that the research was conducted in the absence of any commercial or financial relationships that could be construed as a potential conflict of interest.

## Footnotes

^1^http://www.jwatcher.ucla.edu/
